# Tumor Antigen-Dependent and Tumor Antigen-Independent Activation of Antitumor Activity in T Cells by a Bispecific Antibody-Modified Tumor Vaccine

**DOI:** 10.1155/2010/423781

**Published:** 2011-03-01

**Authors:** Philippe Fournier, Volker Schirrmacher

**Affiliations:** ^1^German Cancer Research Center (DKFZ), Im Neuenheimer Feld 280, 69120 Heidelberg, Germany; ^2^Tumor Immunology Program, DKFZ, Im Neuenheimer Feld 280, 69120 Heidelberg, Germany; ^3^Medical Centre for Immunology and Oncology (IOZK), 50674 Cologne, Germany

## Abstract

New approaches of therapeutic cancer vaccination are needed to improve the antitumor activity of T cells from cancer patients. We studied over the last years the activation of human T cells for tumor attack. To this end, we combined the personalized therapeutic tumor vaccine ATV-NDV—which is obtained by isolation, short *in vitro* culture, irradiation, and infection of patient's tumor cells by Newcastle Disease Virus (NDV)—with bispecific antibodies (bsAbs) binding to this vaccine and introducing anti-CD3 (signal 1) and anti-CD28 (signal 2) antibody activities. This vaccine called ATV-NDV/bsAb showed the unique ability to reactivate a preexisting potentially anergized antitumor memory T cell repertoire. But it also activated naive T cells to have antitumor properties *in vitro* and *in vivo*. This innovative concept of direct activation of cancer patients' T cells via cognate and noncognate interactions provides potential for inducing strong antitumor activities aiming at overriding T cell anergy and tumor immune escape mechanisms.

## 1. Introduction

For decades, treatment of cancer has focused primarily on surgery, chemotherapy, and radiation. Despite significant advances by the introduction of new chemotherapeutic agents and also recently by the clinical introduction of monoclonal antibodies, major limitations of such treatments keep the inability to eliminate the last tumor cell. The offspring of those tumor cells that were not destroyed by the first-line treatment may have a selective advantage, leaving the patient with a recurrence of cancer that is often widespread and resistant to further chemotherapy or radiotherapy. Therefore, more effective therapies are needed. Immunotherapy based on antitumor immune memory is a new modality for cancer treatment. It holds great promise for affecting in a positive way cancer patients' survival with minimal toxicity. 

For over 200 years, active immunotherapy approaches have been used to successfully prevent numerous infectious diseases such as smallpox. These active immunotherapy concepts are now being applied to develop therapeutic cancer vaccines with the intention of treating existing tumors and/or preventing tumor recurrence. In principle, anticancer vaccination (e.g., with autologous tumor cells, peptide vaccines, dendritic cells, idiotypic antibodies, and virus-based vaccines) is a meaningful additional approach for treatment of cancer [1].

Tumor antigens (TAs) of patients' tumor cannot be recognized directly by the patients' T cells. They need first to be processed and to be properly presented by specialized cells that are known as professional antigen-presenting cells (APCs) such as dendritic cells (DCs). TA-presenting DCs then migrate to lymph nodes, where they induce immunity in TA-specific naive T cells. This results in the differentiation into effector T cells—mainly CD8+ cytolytic T cells (CTLs), which are capable of destroying tumor cells expressing the tumor antigen. The response also leads to the generation of TA-specific memory T cells which provide immune protection against tumor recurrence. 

Despite promising results of cancer vaccination obtained in animal tumor models, results of published vaccine trials reveal only a weak clinical response rate with less than 1% for active specific immunization procedures in colorectal cancer patients [2]. At the Surgery Branch of the National Cancer Institute (Bethesda, Maryland, USA), an objective response rate of only 2.6% was reported [3] with various cancer vaccines, even though about 50% of the vaccinated patients had developed CTL killer cells able to specifically recognize and kill tumor cells in vitro.

Difficulties met by vaccination approaches to cancer treatment have been attributed to tumor immune avoidance mechanisms [4]. Tumors employ many escape strategies in order to evade immune attack. These strategies include downregulation of MHC molecules in order to hide from immune recognition [5], expression of inhibitory factors and immunosuppressive cytokines [6–8], including TGF-*β* [9, 10], IL-10 [11], and recruitment of regulatory immune cells CD4+CD25+FoxP3+ Tregs [12], Tr1 cells [13], tolerogenic DCs, and myeloid suppressor cells, including immature macrophages, granulocytes, DCs, and other myeloid cells at earlier stages of differentiation [14, 15]. These immune avoidance mechanisms employed by tumors render the immune system tolerant. This may be responsible for tumor immune evasion as many of the tolerance mechanisms that prevent autoimmunity are the same as employed by tumors to prevent immune destruction [16, 17]. 

In order to develop an effective immunotherapy strategy for metastatic cancer, new approaches are required that not only can create and enhance tumor-specific immunity but can also counteract the ability of the tumor to evade immune destruction. To this end, T cells of the cancer patients need to be educated to attack tumor cells. Naive CD8+ T cells require two distinct signals for activation: signal 1 is provided by engagement of the TCR with its cognate ligand, and signal 2 is provided by interaction of costimulatory receptors with their respective ligands on the APCs [18, 19]. Memory CD8+ T cells, which have been primed to TA, are often anergic and need to be properly reactivated in order to be able to destroy the tumor cells.

The design of an efficient antitumor vaccine may be influenced by an important paradigm shift in the field of immunology regarding the regulation of immunity. A new concept has emerged that proposes that the regulation of immunity and tolerance is not only determined by the specificity of immune T cells as previously thought but also by the context in which the antigens are presented to the immune system [20, 21]. The implications are that, in the absence of appropriate inflammatory reactions, the self- (tumor) antigens presented by APCs will not lead to T cell activation. Since tumors can also produce anti-inflammatory cytokines, they are capable of influencing the immune response by preventing an inflammatory response.

Therefore, successful antitumor immunity will develop only in situations where DCs are processing TAs in the presence of an inflammatory microenvironment (“danger signals”) which is potent enough to also downregulate tumor-mediated immunosuppressive cytokine production. The magnitude and duration of the immune response will be dependent on the extent and quality of the local inflammatory response and will be contained by a variety of existing tolerogenic mechanisms. 

Previous attempts at developing therapeutic cancer vaccines have demonstrated that it is possible to elicit specific immunity against self-tumor antigens [2, 3]. Recent insights on how immunity and tolerance are regulated indicate that the failure of these vaccines in the clinic may be related to the absence of sufficient danger and T cell costimulation signals at the time when tumor antigens are processed by DCs.

In this paper, we highlight some *in vitro* and *in vivo* observations made during the evaluation of a tumor vaccine that we developed in our laboratory. The tumor vaccine of the second generation, modified with bsAb, will be shown to be capable to reactivate memory T cells and to activate nonspecifically naive T cells against the tumor.

## 2. The Autologous NDV-Based Tumor Vaccine

**Figure 1:**
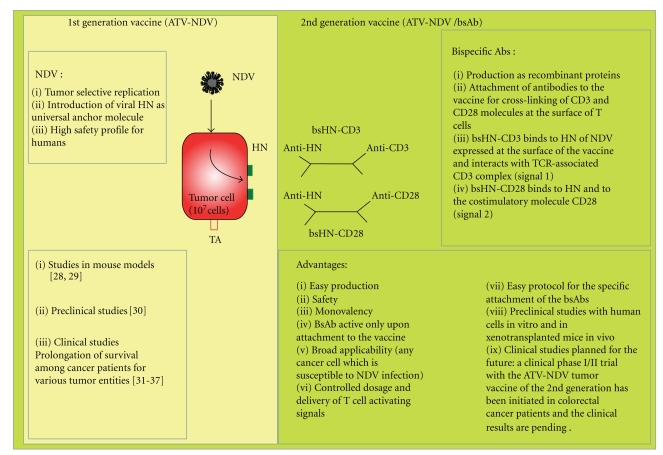
Principles of the NDV-based tumor vaccine of the first and second generation and status of the art (for more details, see the main text).

Over the last 10 years, we have developed and evaluated an autologous tumor vaccine which is first modified by virus infection and which later was modified further by attachment of bispecific antibodies (see Figure 1). The aim was to activate with such a vaccine potentially anergized TA-specific memory T cells and to activate in addition nonspecifically naive T cells to overcome tumor escape variants that may lack TA expression. For virus infection, we chose the avian paramyxovirus Newcastle Disease Virus (NDV) [22]. NDV is one of five species of viruses that are under clinical evaluation [23]. It is a negative strand RNA virus with interesting antineoplastic and immune-stimulating properties [23, 24]. Most remarkable is its capacity to induce strong type I interferon responses by viral protein [24] and RNA [25]. Detection of foreign RNA in the cytoplasm by RIG-I induces an innate antiviral program that initiates the transcription of RNA-responsive genes. The responses involve a multimodal machinery of gene regulation by the Interferon Regulatory Factor (IRF) family of transcription factors [26] and link innate and adaptive immunity [27]. There are 2 generations of NDV-based tumor vaccine: the ATV-NDV and ATV-NDV/bsAb.

### 2.1. First-Generation Vaccine: ATV-NDV

The virus-modified tumour vaccine developed by us for human application consists of virus-infected intact viable and irradiated autologous tumor cells (see Figure 1). The tumor cell infection by NDV is designed to provide the necessary danger signals to elicit antitumor immunity. This strategy is based on preclinical studies in metastatic animal tumours. Antimetastatic effects were observed after local postoperative vaccination with NDV-infected autologous tumour cells [28]. The vaccination activated a tumour-line specific T cell-mediated immune response, which also protected against a second challenge with the same tumour line [29].

Tumor cell infection by NDV was found also in humans to be an efficient and safe way to produce an autologous tumor vaccine (first-generation ATV-NDV) with pleiotropic immuno-stimulatory properties [30]. Promising results based on the prolongation of survival among cancer patients with various tumor entities have already been reported from several clinical phase II trials, including breast cancer [31, 32], colon carcinoma [33, 34], Head and Neck Squamous Cell Carcinoma (HNSCC) [35], and glioblastoma [36]. The antitumor clinical efficacy has been shown also in a randomized study performed among colon carcinoma patients operated for liver metastasis [37].

### 2.2. Second-Generation Vaccine: ATV-NDV/bsAb

The use of NDV during the generation of the ATV-NDV tumor vaccine presents the advantage that, upon infection, the viral hemagglutinin-neuraminidase (HN) protein is expressed on the vaccine cells and can serve as universal anchor molecule for the binding of new ligand proteins. The ATV-NDV can then be combined easily with bispecific antibodies (bsAbs) binding to the HN protein for introducing anti-CD3 and anti-CD28 antibodies at the surface of the tumor vaccine to obtain the NDV-based tumor vaccine of the second generation (see Figure 1). This novel strategy is designed to intensify T cell activation via agonistic anti-CD3 and/or anti-CD28 single-chain antibody reagents (scFv). When suboptimal amounts of anti-CD3 are employed, the combination of TA, anti-CD3, and anti-CD28 should help to intensify TA-induced signal 1 and HN-induced costimulatory signal 2 [38, 39]. Because of virus infection, the ATV-NDV/bsAb vaccine provides a highly inflammatory environment for T cells in the presence of tumor cells. The presence of cell surface bsAbs binding to CD3 and CD28 receptor molecules serves among other effects for augmenting signal strength in T cells to override anergic states of TA-specific T cells. Signal intensity and duration (strength) of TCR stimulation has an impact on setting the balance between adaptive responses and immunopathology [40] and influences induction of T cell activation or anergy [41]. Signal strength, timing, and tuning are also important for T cell costimulation (signal 2). Combining optimal signals 1 and 2 at the surface of the tumor vaccine is expected to generate strong antitumor adults. We showed over the last years that this vaccine acts on TA-specific memory T cells but also on naive T cells via TA-dependent and TA-indepentent pathways. 

## 3. Reactivation of TA-Specific Memory T Cells from Cancer Patients upon Optimal Combination of bsHN-CD3 and bsHN-CD28 with the Vaccine ATV-NDV

**Figure 2:**
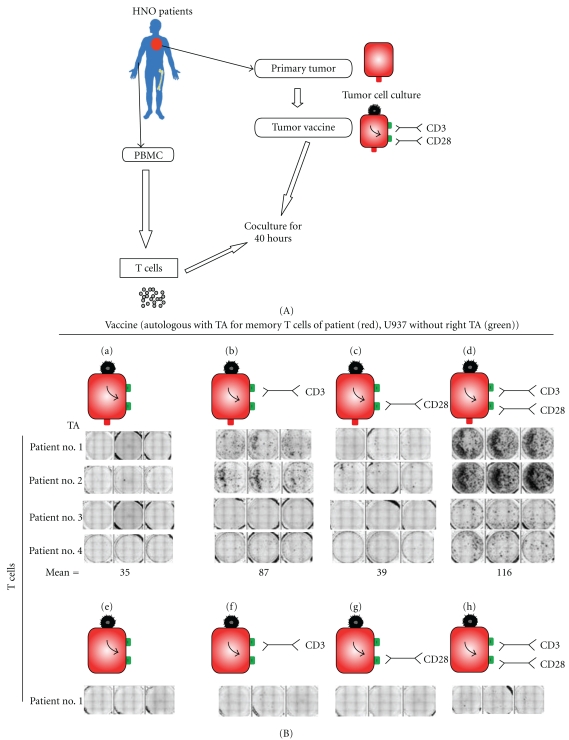
Reactivation of tumor-reactive memory T cells of cancer patients (here HNO cancer patients) by the ATV-NDV tumor vaccine of the 2nd generation. (A) Protocol for the ex vivo stimulation of T cells from cancer patients by the NDV-based tumor vaccine of the second generation in an autologous setting. Purified T cells isolated from tumor-draining lymph nodes (LN) of HNSCC patients were tested for their capacity to be restimulated and to produce IFN-*γ* upon contact with various combination of the ATV-NDV tumor vaccine (generated from the autologous tumor) and bsAbs molecules. For that, they were coincubated for 40 hours with the indicated autologous vaccine formulations (tumor) (*n* = 4). For specificity control, the unrelated human promonocytic tumor cell line U937 (U937) was modified identically and used to reactivate the patients' T cells. (B) IFN-*γ* ELISPOT results from A. Mean: mean number from triplicates of 4 patients spot forming T cells per 1 million cells. Each patient's T cells were stimulated with four different formulations of either autologous (a–d) or heterologous (e–h) vaccine. T cells of patients no. 2, no. 3, and no. 4 also did not react to the heterologous vaccine (data not shown) (adapted from [42]).

The ATV-NDV tumor vaccine of the second generation was capable of reactivating anergic T cells from tumor-draining lymph nodes of cancer patients. This was revealed via a modified short-term IFN-*γ* ELISpot assay which we established for reactivation of cancer-reactive memory T cells. As shown in Figure 2(A), from four different head and neck squamous cell carcinoma (HNSCC) patients, primary tumor cells were expanded *in vitro,* and, from each tumor line, autologous tumor vaccines were prepared as a vaccine (through irradiation, infection by NDV, and loading with the bsAbs). These vaccines were finally combined with T cells isolated from tumor-draining lymph nodes from the corresponding HNSCC patients. The data from Figure 2(B) show a strong IFN-*γ* response only in that group where T cells were stimulated with autologous TAs together with anti-CD3 and anti-CD28 signals. These responses required HNSCC-derived TAs. Another tumor line (the heterologous promonocytic U937 cell line) modified following the same way could not reactivate a short-term memory response from the patients' T cells (Figure 2 (B), lower part).

We conclude

- that tumor-reactive memory T cells from draining lymph nodes of HNSCC patients could not be activated with the first-generation vaccine ATV-NDV,
- that the cells, however, could become efficiently reactivated using the same vaccine together with optimized signals 1 and 2, and
- that a similarly modified vaccine from an unrelated tumor cell line was not capable to elicit such a memory T cell response.

The fact that the ATV-NDV vaccine without bsAbs was not capable to reactivate a response might be explained by an anergic state of the tumor-draining lymph node-derived T cells.

## 4. Activation of Antitumor Activity from Naive T Cells by the Second-Generation Vaccine

**Figure 3:**
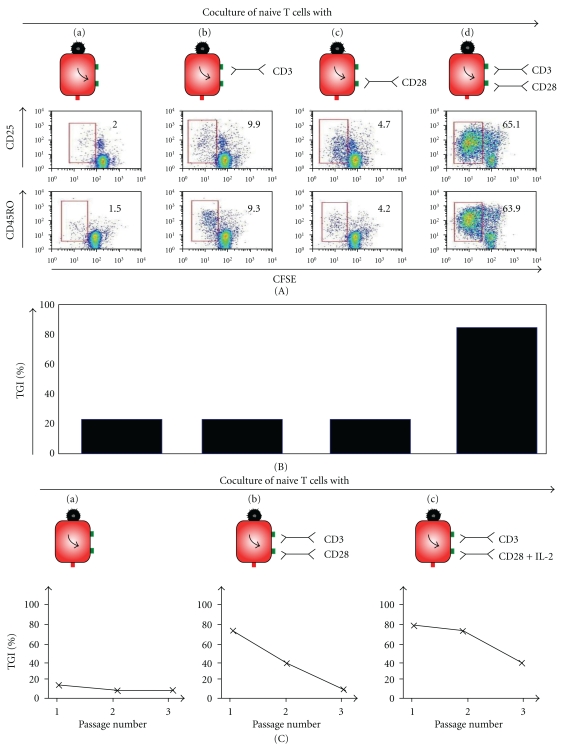
Activation of naive T cells towards tumor cells by the NDV-based tumor vaccine combined with bsAbs in different combinations.

The tumor vaccine ATV-NDV/bsAb was shown to have also an effect on naive T cells which were obtained from total T cells of normal healthy donors by removal of the CD45RO+ cells. This was observed by analyzing the activation of the naive T cells towards tumor cells after 6 days of incubation with various vaccines. Figure 3(A) shows CD25 and CD45RO expression on naive T cells upon coincubation with various NDV-based tumor vaccines. 1 × 10^5^ naive T cells from a normal healthy donor were labeled with CFSE. They were then coincubated with 1 × 10^4^ irradiated MCF-7 cells which were modified with 100 HU NDV Ulster and with suboptimal bsHN-CD3 (500 pg/well) and 84.4 U/well bsHN-CD28 (d). Naive T cells, which came in contact only with the vaccine (a) or with the vaccine loaded with each of the constructs separately (b and c) served as controls. After six days, the cells were harvested, blocked with Endobulin, and then stained with anti-CD25-APC (a) or anti-CD45RO-PE (b) before being analyzed on the FACS. Dead cells were excluded by propidium iodide staining. The red boxes indicate actively dividing (CFSE low) cells expressing CD25 or CD45RO. The frequencies are indicated as numbers in % (adapted from [42]). We observed a strong activation only when both bsAbs (bsHN-CD3 and bsHN-CD28) were added to the tumor vaccine (see Figure 3(A), (d)). Figure 3(B) shows that tumor growth inhibition induced in naive T cells upon coincubation with various NDV-based tumor vaccines. In order to test the effectiveness of the new vaccine strategy to activate *in vitro* naive T cells, an *in vitro* assay that we called tumor neutralization assay (TNA) was developed. It consists of an adherent tumor cell monolayer (here human MCF-7 breast cancer cells) in a cell culture plate in which *γ*-irradiated NDV-modified vaccine cells, fusion proteins, and naive T cells of a healthy donor as effector cells are brought into contact. During the incubation period of five to seven days, the vaccine cells activate the effector cells which increase their cytotoxic potential. During the effector phase, the “bystander” tumor cells are lysed, or their growth is inhibited. This results in a decrease in the number of live tumor cells in monolayer (when compared to the controls). After removal of the nonadhering remaining effector cells, the number of surviving tumor cells can be quantified with MTS as dye reagent for measuring the amount of viable tumor cells per well. We observed that the tumor vaccine with the 2 bound bsAbs bsHN-CD3 and bsHN-CD28 was by far the most efficient in this assay among all the combinations of tumor vaccine and bsAbs tested (Figure 3(B)). We also observed that the extent of T cell-mediated tumor growth inhibition *in vitro* depended on an optimal amount of bsHN-CD3 and bsHN-CD28 present at the surface of the tumor vaccine (data not shown, see [42]). Titration curves revealed for each of the recombinant proteins, upon attachment to the vaccine, a low dose optimum, of T cell stimulating or costimulating activity [42].

An important aspect of T cell effector activity relates to its duration. To test this, we activated purified T cells once by coculture with the second-generation vaccine, performed the TNA, and then repeatedly transferred the T cells from the suspension above the destroyed monolayer onto fresh tumor monolayers and followed their destruction thereafter. In these ways, live adherent MCF-7 monolayers were coincubated in 96-well plates with MCF-7-NDV vaccine cells, purified T cells, a suboptimal dose of bsHN-CD3 (1 *μ*g per 10^7^ vaccine cells; signal 1), and one of the following costimulatory fusion proteins: bsHN-CD28 (signal 2a) or tsHN-IL-2-CD28 (signal 2a-2b), each at a concentration of 84 U per 10^7^ vaccine cells (passage 0). T cells activated in the presence of suboptimal bsHNCD3 alone (negative controls) showed no tumor growth inhibition in this assay (data not shown). For serial passages, the same conditions were performed in parallel using a 6-well TNA format with identical protein concentrations (TNA passage plates). After 7 days, the TNA test plates were developed using MTS as reagent to obtain the value of tumor growth inhibition as described in [43]. The cells from the TNA passage plates were harvested, washed, and transferred for another 3 days onto fresh MCF-7 monolayers, either in 96-well TNA test plates (passage 1) or in 6-well TNA passage plates for another round (passage 2). The results (Figure 3(C)) revealed a much longer duration of bystander antitumor activity in T cells activated by the vaccine bound bsHN-CD3 and bsHN-CD28 (group b) than in T cells activated by the vaccine alone (group a). We have constructed also a trispecific recombinant fusion protein (tsHN-IL-2-CD28) in which the cytokine interleukin-2 was linked in between the anti-HN and the anti-CD28 scFv binding sites [42]. Since we had observed that vaccine-bound IL-2 can deliver costimulatory signal via CD25 (the IL-2 receptor alpha chain) to T cells, we were then interested to test whether the combination of costimulatory signals delivered via anti-CD28 and via IL-2 might have an advantage. As can be seen from the results in Figure 3(C), group c, this type of modified vaccine induced antitumor effecter activity of the longest duration. Expressed quantitatively, a simultaneous introduction of costimulatory signals via CD28 (signal 2a) and IL-2 receptor (signal 2b) led to a 20–40% increased bystander antitumor activity in passage 1 (days 7–10) and passage 2 (days 10–13) when compared to a tumor vaccine with only one costimulatory signal (for more details, see [42]).

## 5. Human PBMC Preactivated In Vitro by the NDV-Based Tumor Vaccine of the Second Generation Shows In Vivo Therapeutic Efficiency upon Adoptive Transfer to Tumor-Bearing Mice

**Figure 4:**
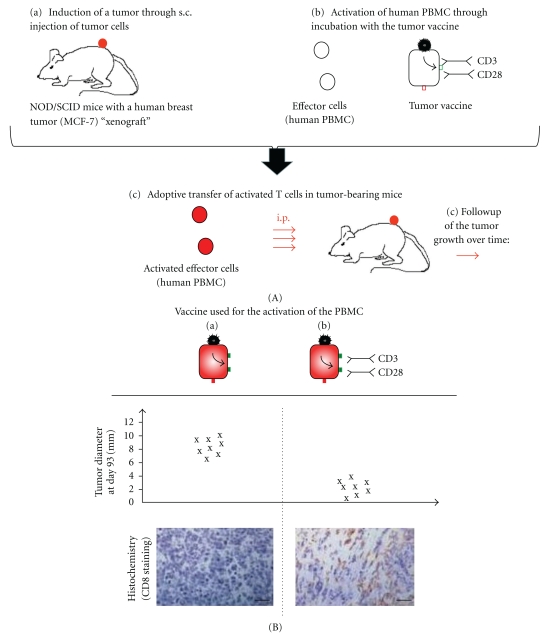
*In vivo* antitumor activities of human PBMC preactivated *in vitro* by the NDV-based tumor vaccine of the second generation. (A) Experimental protocol. MCF-7 tumor cells were injected subcutaneously into NOD/SCID mice (*n* = 8 per group). The mice were kept until palpable tumors were established (6–8 mm in diameter) (a). Then, at days 1, 4, and 7, the mice were treated by i.p. injection of 10^7^ PBMC which were preactivated *ex vivo* for 3 days by the NDV-based tumor vaccine of the first or second generation obtained using the MCF-7 cell line (b). Tumor growth was then monitored over time (c). (B) Tumor diameter and representative immunohistochemistry images of tumor tissue sections. Tumor-bearing mice were treated with PBMC activated with MCF7-NDV (left) or with PBMC preactivated with MCF-7-NDV loaded with bsHN-CD3 and bsHN-CD28 (right) and were analyzed over time for tumor growth, and data at day 93 is represented (top). At this time point, some mice were sacrificed, and the tumor sections were stained with mAbs against the human CD8 antigen and analyzed by fluorescence microscopy (bottom). Scale bar, 100 *μ*m (adapted from [42]).

To study possible immunotherapeutic effects of vaccine-activated PBMC *in vivo*, we used a NOD/SCID mouse model which allows outgrowth of human MCF-7 breast cancer cells [44]. For therapy, PBMCs from a healthy donor were preactivated with an MCF-7-NDV tumor vaccine loaded or not with bsHN-CD3 and bsHN-CD28 [42]. The preactivated cells were then transferred via intraperitoneal injections into mice with established MCF-7 xenotransplants (Figure 4(A)). PBS-treated control mice showed progressive tumor growth, as did tumors from mice treated with PBMC preactivated with MCF-7-NDV tumor vaccine. The tumors of mice, however, treated with PBMCs, which were preactivated with the NDV-based tumor vaccine of the second generation, started to regress 51 days after adoptive cell transfer and showed at day 93 much smaller tumor diameters when compared to tumors of mice treated with the NDV-based tumor vaccine of the first generation (i.e., without the 2 bsAbs) (see Figure 4(B)). Notably, treatment of mice with supernatants from PBMC cultures preactivated with the NDV-based tumor vaccine of the second generation did not lead to tumor regression [42]. Immunohistochemical stainings of tumors that underwent regression revealed a heavy infiltration by both human CD4+ and CD8+ T cells (Figure 4(B)), a certain percentage of which were in an activated state (CD69 positive) and exhibited a memory phenotype (CD45RO positive) [42]. In contrast, tumor sections from mice treated with PBMC preactivated with the MCF-7-NDV tumor vaccine (Figure 4(B)) or with PBS (data not shown) revealed no tumor-infiltrating T cells [42]. 

In summary, human naive T cells upon stimulation with the second-generation tumor vaccine and upon transfer *in vivo* can—after a certain lag period—infiltrate human tumor tissue and mediate tumor regression.

## 6. Conclusion and Perspectives

The main question we address in this paper: how can we exploit maximal antitumor activity from T cells of cancer patients through antitumor vaccination?

Over the last decades, many types of vaccines have been developed towards this goal, including synthetic peptides, “naked” DNA, dendritic cells, or recombinant viruses. Our approach is based on the use of patient-derived tumor cells which are irradiated, modified by infection with NDV, and coupled with bispecific Abs in order to introduce new ligands for T cell activation at the surface of the tumor vaccine.

**Figure 5:**
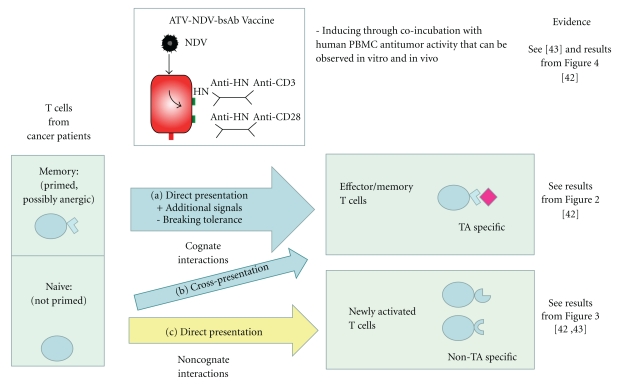
Cognate and noncognate antitumor mechanisms induced by the NDV-based tumor vaccine of the second generation (ATV-NDV/bsAbs). The different mechanisms of T cell activation against tumor suggested explaining the efficacy of the ATV-NDV/bsAb in inducing antitumor activity in T cells are highlighted. (a) Direct antigen presentation of TA to memory T cells. Addition of danger signals via NDV infection of the tumor cells during the elaboration of the tumor vaccine induces a reactivation of the antitumor reactivity in TA-specific memory T cells [32]. This mechanism is intensified by the addition of the 2 bsAbs introducing binding activities to CD3 and CD28 at the surface of the tumor vaccine (see Figure 2). + signal intensification via bsAb (memory T cells). (b) Cross-presentation via DCs *in vivo* (naive T cells). (c) Direct polyclonal activation this pathway is highlighted in this manuscript.

We observed that the NDV-based autologous tumor vaccine of the second generation can be used for reactivation of apparently anergic memory T cells from tumor-draining lymph nodes of individual cancer patients. The danger and costimulatory signals introduced to the vaccine through virus infection as well as additional signal 1 and 2 at the surface of the tumor vaccine appear necessary in cancer patients whose T cells exhibit a high degree of unresponsiveness to stimulation to TA. The underlying mechanism explaining such antitumor activity is suggested to rely on direct presentation of autologous TAs (cognate interactions with memory T cells (see Figure 5)) and on augmentation of signal intensity by bsAbs. 

The absence of any response in the short-time Interferon-*γ* Elispot (see Figure 2, lower part) during the coincubation of patient T cells and heterologous bsAb-modified tumor cells might be explained by the absence of autologous TA. 

What is also interesting is the capacity of the NDV-based tumor vaccine of the 2nd generation to induce antitumor activity in naive T cells. Such activation of naive T cells requires, however, a longer time period than that of memory T cells. The proposed mechanism is direct T cell activation via noncognate interactions with the vaccine/bsAb leading to induction of strong bystander antitumor activity (see Figure 5 and Table 1). These latter observations suggest that T cells can be activated to exert antitumor activity by such a new tumor vaccine in a TA- and MHC-independent pathway, similar to cells of the innate immune system. This new mechanism may become an important safeguard against tumor immune escape.

The cells of the adaptive immune system utilize somatically rearranged receptors to recognize antigens. By contrast, cells of the innate immune system primarily use germline-encoded receptors to defend against infected or transformed cells. Interestingly, cells of the adaptive immune system can express also some of these germline-encoded receptors [45].

Can T cells, in similarity to cells of the innate immune system, sense danger? The first published “danger model” of immunity [46] proposed only one mechanism for immune recognition of danger that perceived by DCs upon release of cellular contents following necrosis of a diseased cell in its neighbourhood. This model predicts a superior effect of a lytic as opposed to a nonlytic virus in the treatment of tumors, because tumor cells necrotically destroyed by the virus would be phagocytosed and perceived as dangerous by DCs. The DCs would (i) process TAs, (ii) become activated, and (iii) present processed TA peptides to T cells for cognate interaction and immune response induction.

Another recent model [47] suggests that T lymphocytes themselves correlate danger signals to antigen. This hypothesis associates danger also with nonlytic viruses (as NDV Ulster) if these are upregulating danger signals in their host cells. Such an event will quickly cause its host cells to be killed by the immune system. Killed infected tumor cells are likely to result in TA being presented by DC along with potent costimulation. Recently, it was shown that dsRNA in the apoptotic bodies of virus-infected dead cells is recognized by CD8 alpha+ DCs that have high expression of toll-like receptor 3 (TLR-3) [48]. This promotes cross-priming of T cells to virus-infected cells [49].

Recent data support a role for CD8+ T cells in innate immune responses, independent of TCR specificity. Under certain circumstances, antigen non-specific TCR-independent responses of CD8+ T cells play a beneficial role in controlling tumors [50]. Marsland et al. [51] showed that innate signals driven by DC can compensate for the absence of PKC-Φ, which is important for TCR signalling, during CD8+ T cell effector and memory responses *in vivo*.

The discovery of toll-like receptors (TLRs) and more recently of cytosolic innate immune sensors such as RIG-I-like receptors (RLRs) and nod-like receptors (NLRs) as components that recognize structures of “danger” such as pathogens has greatly advanced understanding of how innate immune response can be triggered and can prime antigen-specific adaptive immunity. Cytotoxic CD8+ T cells and T helper 1 (Th1) cells are central to effective immune responses against tumor. However, clinical trials with cancer vaccines have shown the weakness of responses observed among the treated cancer patients, although cytotoxic activities specific for the targeted TA were detected *in vivo* [3].

 A key determinant in the induction of a strong and efficient cellular immune response against tumors seems to be, in addition to a broad repertoire of TA presented by the tumor cells used as vaccine, the recognition of the TA as “nonself.” Such TAs have been termed “unique” (individually tumor specific) and have a potential to serve as tumor rejection antigen. The superiority of autologous tumor vaccines among tumor vaccines evaluated in randomized clinical studies [52] suggests that unique tumor antigens are indeed particularly important in generating responsive T cells for a therapeutic effect. Restimulation of TA-specific memory T cells and activation of naive T cells may explain the strong antitumor potential of the NDV-based and bsAb-modified tumor vaccine.

CD8+ T cells have been implicated not only in antigen-dependent but also in antigen-independent antitumor responses. Self-reactive CD8+ T cells expressing high levels of NKG2D induce killing of target cells using a redirected lysis assay [53]. Furthermore, activation of CD44hi CD8+ T cells using IL-2 resulted in significantly higher levels of killing of a syngeneic target compared with CD44loCD8+ T cells [54]. Expression of the NKG2D ligand Rae-1*δ* resulted in increased killing of the syngeneic targets. As the expression of Rae-1*δ* is associated with tumors, it seems plausible that a subset of innate CD8+ T cells activated with IL-2 could directly lyse tumors using NKG2D, independent of TCR specificity. Another report characterized CD8+ T cells in cancer patients receiving IL-12, showing that a subset of CD8+ T cells expanded and displayed enhanced MHC nonrestricted cytotoxicity *in vitro* [55]. This suggests that cytokines can stimulate subsets of CD8+ T cells, independent of TCR signalling, to lyse tumors. In this context, it is interesting to see an example of clinical application: the transfusion of intentionally mismatched donor lymphocytes in high-risk chemotherapy-resistant patients with metastatic solid tumors and haematological malignancies [56].

Danger signals and T cell costimulation have great therapeutic potential but need to be optimized and controlled. Unfortunately, these same types of innate antigen-independent responses might be harmful when they are self-reactive. The risk factor for induction of autoimmune disease (see Table 1) has been discussed in more detail in a recent editorial [57]. 

In summary, the tumor vaccine ATV-NDV/bsAb shows dual activity: (i) it can activate TA-specific memory T cells from cancer patients, and (ii) it can generate antitumor activity from naive T cells. A strategy harnessing both arms of the immune system—innate (based on noncognate interactions) and adaptive (relying on cognate interactions)—holds great potential for clinical applications in cancer patients.

Our findings open new ways of investigations to manipulate T cell activity against tumor cells and to exploit the full power of the immune system through reactivation of tumor antigen-specific memory T cells and through de novo activation of naive T cells. These could, for example, be based on the combination of new recombinant immunostimulatory molecules such as bi- or trispecific antibodies or immunocytokines with vaccines which have already proven clinical effectivity.

## Figures and Tables

**Table 1:** Summary of the types of interactions, mechanisms, characteristics of the immune responses induced by the ATV-NDV/bsAb vaccine in memory and naive T cells and the eventual danger of autoimmunity (see the main text for more details).

Vaccine + bsAbs	Interactions T cells-tumor cells	Mechanisms	Response characteristics	Autoimmunity
TA + bsCD3 + bsCD28	Cognate	Restimulation (memory)/TA cross-presentation via DCs (naive) *⇒* TA-specific T cell activation	Long-term effect systemic	Low risk

bsCD3 + bsCD28	Noncognate	Provision of inflammatory and danger signals *⇒* Non-TA-specific T cell activation	Transient, local	High risk
